# The Varieties of Anæmia in Chronic Tuberculosis

**Published:** 1908-07-18

**Authors:** 


### Medicine.

### THE VARIETIES OF ANÆMIA IN CHRONIC TUBERCULOSIS.

The appearance of the face in patients suffering from chronic tuberculosis is very deceptive as a guide to the condition of the oxygen-carrying power of the blood. Sometimes there is pallor without real anæmia; sometimes the face has a good colour although the quantity of the blood in the body is less than normal. If reliance is placed upon a mere enumeration of the red corpuscles and upon an estimation of the percentage of hæmoglobin, an erroneous conception of the patient's blood-condition may be formed. It is necessary to take into account the variations in the total mass of blood at the same time as the red corpuscles are counted if tuberculous or any other anæmia is to be appreciated properly, and if certain results of blood counts are to be correctly interpreted.

It is clear that anæmia is in proportion to the total quantity of oxyhæmoglobin in the whole organism rather than to the numbers of corpuscles in each cubic millimetre of blood. That which matters most is the amount of oxygen that the blood is able to carry to the tissues, and this amount depends upon the mass of the blood and its functional value.

Unfortunately, it is very difficult to measure the total volume of blood in practice. The carbon monoxide method of Haldane and Lorrain Smith can seldom be applied. Nevertheless, there are a certain number of points which afford indirect assistance in arriving at a conclusion as to the blood-volume in a given case. The blood-pressure is one of these factors. If all other conditions remained constant—the force of the cardiac action, the degree of peripheral vaso-dilatation, and so forth—an increase in the total volume of the blood would cause some rise of pressure, and vice versa. The colour of the skin is also of some assistance, if it is interpreted in terms of the blood count. If the number of red corpuscles per cub. mm. and the percentage of hæmoglobin are not much below normal, pallor of the face indicates diminution in the total blood in the body. A third factor is the variation in the patient's weight: very roughly speaking, when the weight rises the mass of blood is increasing; when the weight falls, the blood mass diminishes along with the other tissues and body fluids.

Dr. Marcel Labbé has laid considerable stress upon all these separate points, and, by paying attention to the number of red corpuscles per cub. mm., the percentage of hæmoglobin, the arterial blood pressure, and the colour of the skin and mucous membranes, he is led to distinguish three distinct types of anæmia in chronic tuberculosis, namely: (1) *Anæmia with Ochrodermia.*—Pallor with real diminution in the amount of blood. (2) *Anæmia without Ochrodermia.*—Real diminution in the amount of blood in a patient with a deceptive redness of the complexion. (3) *Ochrodermia without Anæmia.*—Pallor without any marked diminution in the red cells or hæmoglobin as counted in the ordinary way; although the total volume of blood in the body is below normal.

### 1. Anæmia with Ochrodermia.

The majority of tuberculous patients, especially those in whom there is pyrexia, exhibit an ochrodermic facies together with real anæmia. Their appearance is well known. Pallor and emaciation are striking. The features are drawn, the eyes hollow, the cheeks sunken. The skin is whitish or whitish yellow, with the least tint of rose colour in the face. The ears are thin and translucent. The conjunctiva, the buccal mucosa, and the palate are all pale. As regards the blood, the number of red cells per cub. mm. is diminished; sometimes greatly so, but usually only to a moderate extent. In spite of the pallor, it is not uncommon to find 4,000,000 red cells per cub. mm. The percentage of hæmoglobin, on the other hand, is always much below normal. The colour index is therefore invariably low—0.6 or 0.5, for example. The blood picture is similar to that of severe chlorosis. The arterial blood pressure is below normal; instead of being 125 mm. of mercury, it may be 110, 100, or even 90, as determined by the modified Riva Rocci method.

There is not only a diminution of red cells and hæmoglobin per cub. mm. of blood, but there is also a great diminution in the total mass of blood in the body. The functional value of the blood is really much more reduced than would be indicated by enumeration of the red cells only. The four characteristics of this type of anæmia are: Diminution in red cells, greater diminution in hæmoglobin, fall in blood pressure, and pallor of the skin.

As a general rule, this ochrodermic anæmia indicates a pyrexial condition of tubercle, whereas patients who are tuberculous, but who are non-anæmic and non-ochrodermic, are usually apyrexial.

### 2. Anæmia without Ochrodermia.

Although pallor is often a striking feature in chronic tuberculosis, there are a certain number of cases in which there is little or no decolouration of the skin. The face may be thin, the features drawn, the expression tired, but the skin keeps a rose colour and the mucous membranes do not appear pallid; there may even be a certain degree of actual reddening from congestion. At first sight these patients might easily be regarded as not anæmic. If, however, the blood is examined, it often exhibits real anæmia, and the red corpuscles may be down to 4,000,000 or 3,000,000, or even 2,750,000 per cub. mm., notwithstanding the good colour of the face. The percentage of hæmoglobin is also diminished, though much less so in proportion to the red cells than it is in the ochrodermic anæmic cases. The colour index may therefore be very little below 1, seldom coming below 0.8, and often exceeding 0.9.

The blood pressure is much more nearly normal than it is in the preceding type of case. Notwithstanding the fact, therefore, that the number of red corpuscles per cub. mm. is considerably lower than it may be in the anæmic ochrodermic patients, the degree of actual anæmia is much less. The volume of the blood in the body is probably not very much below normal, but the blood is considerably diluted. This form of anæmia without ochrodermia is observed in very diverse tuberculous conditions, sclerosing or caseous, pyrexial or apyrexial.

3. Ochrodermia without Apparent Anæmia.

Lastly, there are the cases of chronic tuberculosis in which, notwithstanding a very pale tint of the face and skin and an appearance of anæmia, blood examination reveals no hæmatic insufficiency. There may be little or no diminution in the number of red corpuscles per cub. mm., little or no fall in the percentage of hæmoglobin, and the colour index may be normal. Moreover, except that the blood pressure is below normal, there are none of the general signs of anæmia such as pulmonary hæmic murmurs, and the like.

This type is met with in a fair number of cases of chronic tuberculosis; but although at first sight it might seem that the blood counts indicate a normal condition, the blood is not really normal. Its total volume is less than it should be, as indicated during life by the diminution in blood pressure and the colour of the face, and as proved at autopsy by the exsanguineous appearance and diminished bulk of the viscera. These are the cases in which, generally in the absence of pyrexia, the hæmolytic action of the tuberculo-toxins has not been great, and in which the reduction in the total blood mass under the influence of insufficient alimentation has been masked. The same type of blood change is brought about by sudden removal of large quantities of fluid from the body by profuse diarrhœa or by copious perspirations.

It is, indeed, sometimes observed that the numbers of red corpuscles per cub. mm. may be even in excess of normal. It is quite clear that, to appreciate properly the degree of an anæmia, one must not be content with merely enumerating the red cells. Blood pressure and skin colour must also be taken into consideration. Blood examination should never be limited to either the red cell enumeration or the hæmoglobin estimation separately; both should be determined, and the colour index calculated. Proportional diminution of red cells and of hæmoglobin may be produced by simple dilution of the blood, whereas diminution in the colour index always indicates that destruction is taking place.

This distinction between the three chief forms of tuberculous anæmia is not merely of theoretical interest; it has a practical significance in treatment. Iron and arsenic do most good in the cases in which there has been destruction of corpuscles—groups 1 and 2; the pallid patients of the third group do not receive the benefit from iron that one might otherwise have expected.

